# C1q/TNF-related protein-9 ameliorates hypoxia-induced pulmonary hypertension by regulating secretion of endothelin-1 and nitric oxide mediated by AMPK in rats

**DOI:** 10.1038/s41598-021-90779-2

**Published:** 2021-05-31

**Authors:** Qiaoyan Jin, Hui Su, Rui Yang, Yanzhen Tan, Buying Li, Wei Yi, Qianqian Dong, Haifeng Zhang, Wenjuan Xing, Xin Sun

**Affiliations:** 1 Department of Pediatrics, Xijing Hospital, Fourth Military Medical University, 127 Changlexi Road, Xi’an, 710032 China; 2 Department of Geriatrics, Xijing Hospital, Fourth Military Medical University, Xi’an, 710032 China; 3 Department of Cardiovascular Surgery, Xijing Hospital, Fourth Military Medical University, Xi’an, 710032 China; 4 Teaching Experiment Center, Fourth Military Medical University, Xi’an, 710032 China; 5 School of Aerospace Medicine, Fourth Military Medical University, 169 Changlexi Road, Xi’an, 710032 China

**Keywords:** Molecular biology, Diseases

## Abstract

Injury/dysfunction of the endothelium of pulmonary arteries contributes to hypoxia-induced pulmonary hypertension (HPH). We investigated whether C1q/tumor necrosis factor-related protein-9 (CTRP9), a newly identified cardiovascular agent, has protective roles in the development of HPH. HPH was induced in adult male rats by chronic hypobaric hypoxia. CTRP9 overexpression by adeno-associated virus (AAV)-CTRP9 transfection attenuated the increases in right ventricular systolic pressure, right ventricular hypertrophy index, and pulmonary arterial remodeling of rats under hypoxia. Importantly, CTRP9 overexpression improved endothelium-dependent vasodilation in pulmonary arterioles in HPH rats. CTRP9 overexpression enhanced expression of phosphorylated 5′-adenosine monophosphate-activated protein kinase (p-AMPK) and phosphorylated endothelial nitric oxide synthase (p-eNOS), and reduced phosphorylated extracellular signal-regulated protein kinase (p-ERK1/2) expression in pulmonary microvascular endothelial cells (PMVECs) of HPH rats. In cultured PMVECs, CTRP9 not only preserved the decrease of AMPK and eNOS phosphorylation level and nitric oxide (NO) production induced by hypoxia, but also blocked the increase in hypoxia-induced ERK1/2 phosphorylation level and endothelin (ET)-1 production. Furthermore, the effects of CTRP9 were interrupted by inhibitors or knockdown of AMPK. CTRP9 enhances NO production and reduces ET-1 production by regulating AMPK activation. CTRP9 could be a target for HPH.

## Introduction

Pulmonary hypertension (PH) is a progressive (and potentially fatal) pathophysiologic disorder. PH involves multiple clinical conditions and can be secondary to cardiovascular and respiratory diseases^1^. Hypoxia-induced pulmonary hypertension (HPH) is PH associated with lung diseases and/or hypoxia^2^.

PH treatment is quite challenging and that of high altitude-related HPH even more so^2^. However, the treatment strategy for HPH is, in general, in accordance with that for PH. The long-term clinical efficacy of such a strategy is not known and the cost can be considerable. Hence, development of novel approaches to improve the worsening outcome and shorter survival observed in HPH is crucial.

The “pulmonary vascular endothelium” refers to the innermost layer of a blood vessel along the route of the heart and lungs. It is crucial for the homeostasis of the pulmonary circulation^3^. Hypoxemia arising from hypoxia stimulates the endothelium of pulmonary arteries directly, and results in impairment and dysfunction of the endothelium as characterized by the aberrant expression and secretion of vasoactive molecules^4,5^. In general, this action gives rise to pulmonary vasoconstriction and pulmonary-artery remodeling, and initiates PH development^6^. Hence, new approaches for HPH treatment should attempt to amend such endothelial dysfunction.

C1q/tumor necrosis factor-related protein-9 (CTRP9) is a novel member of the adipokine family^7^. CTRP9 is involved in lipid metabolism^8^ and cardiovascular protection^9^. CTRP9 can dilate arteries through up-regulation of nitric oxide (NO) expression from the endothelium^10^ as well as protection against endothelial impairment and vascular remodeling^11^. However, its role in regulating dysfunction of the pulmonary vascular endothelium is not known. Considering the pivotal role of dysfunction of the pulmonary endothelium in HPH pathogenesis, we hypothesized that CTRP9 could relieve HPH by modifying pulmonary endothelial functions. We tested the effects of CTRP9 on the production of the vasoactive substances nitric oxide (NO) and endothelin (ET)-1 in vivo and ex vivo.

## Results

### Decreased serum levels of CTRP9 were associated with HPH development

**Figure 1:**
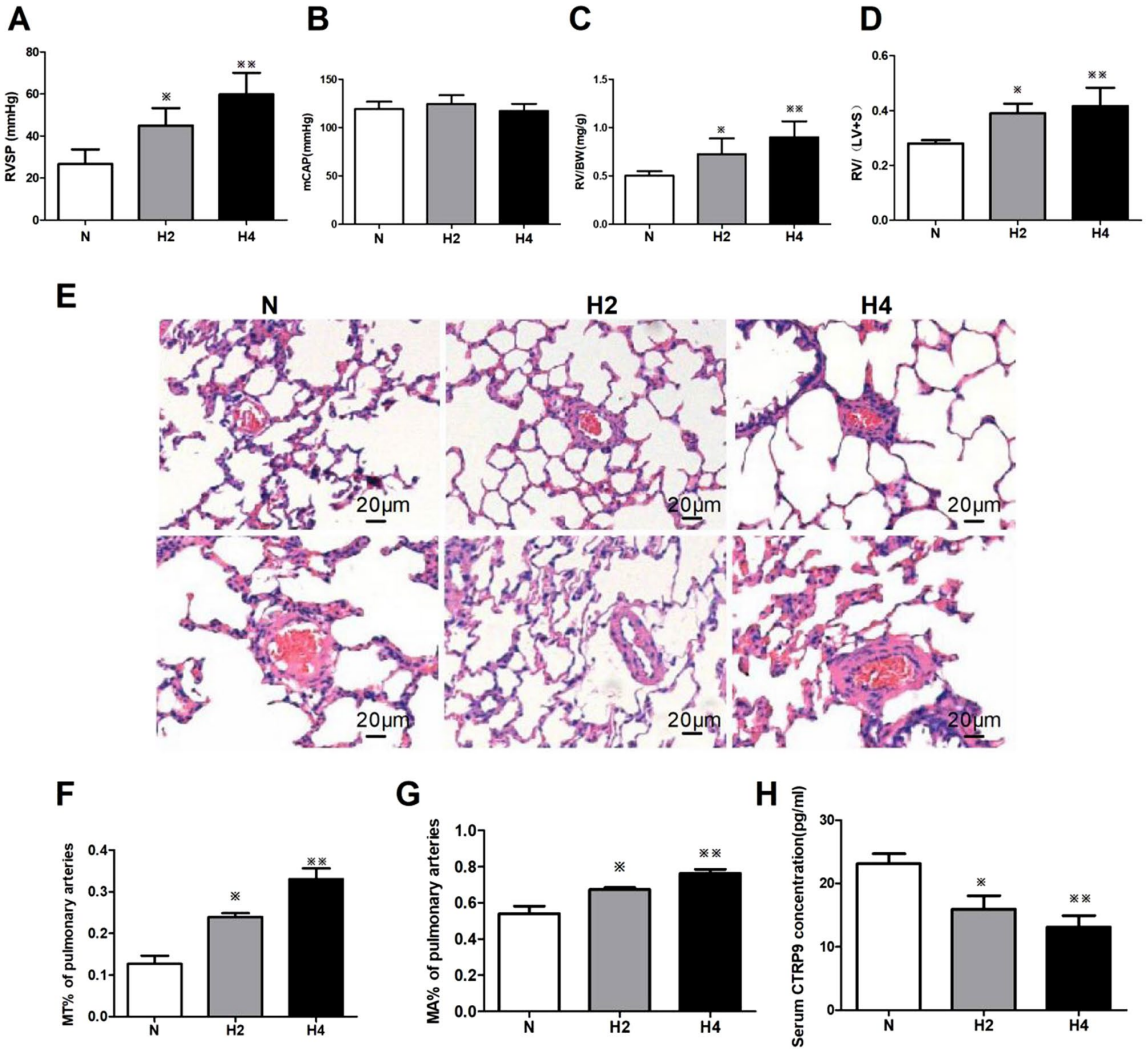
Hypoxia induced cardiopulmonary remodeling and reduction of serum CTRP9 content in rats. (**A**–**D**) Quantitative data of cardiopulmonary hemodynamics, including right ventricular systolic pressure (RVSP, **A**), mean carotid artery pressure (mCAP, **B**), and right ventricular hypertrophy index (**C**, **D**), are presented. € Paraffin-embedded sections of rat lungs were assessed for pulmonary arterial remodeling by staining (hematoxylin & eosin; × 200 magnification). Scale bar = 20 μm. (**F**, **G**) Blinded quantitative analyses of MT% and MA% of peripheral pulmonary arteries (30 vessels/3 sections from one animal, 30–100 μm in diameter) were done using Image-Pro Plus. (**H**) CTRP9 concentrations in the serum of rats suffering hypoxia or not (n = 6). Data are the mean ± SEM. ^*※*^*P* < 0.05, ^*※※*^*P* < 0.01 vs. normoxia group. *N* normoxia for 4 weeks, *H2* hypoxia for 2 weeks, *H4* hypoxia for 4 weeks.

In contrast with the normoxia group, after hypoxia exposure, the right ventricular systolic pressure (RVSP) (Fig. 1A), right ventricular hypertrophy index (RVHI) (Fig. 1C,D), pulmonary-arterial remodeling (Fig. 1E), percentage medial-layer thickness (MT%) and percentage medial-layer area (MA%) of rats in groups H2 and H4 increased significantly (Fig. 1F,G). The extent of the increase in group H4 was greater than that in group H2, but the difference was not significant. Hypoxia had no obvious influence on systemic systolic pressure (Fig. 1B). Intriguingly, the serum CTRP9 concentration decreased significantly during hypoxia, and the extent of this reduction increased over time (Fig. 1H). These results suggested a correlation between a reduced CTRP9 concentration in serum and HPH development.

### CTRP9 overexpression alleviated HPH

In order to investigate further the correlation between a decreased serum concentration of CTRP9 and HPH development. CTRP9 was overexpressed in rats through intra-tracheal instillation of adeno-associated virus (AAV) followed, 3-weeks later, with creation of a HPH model. The effects of AAV transfection in different groups were determined through green fluorescent protein (GFP) expression. GFP expression was approximately identical in the four groups (data not shown), which suggested an equal effect of transfection of AAV-control or AAV-CTRP9 in the lung tissue of rats under hypoxic or normoxic conditions.

**Figure 2:**
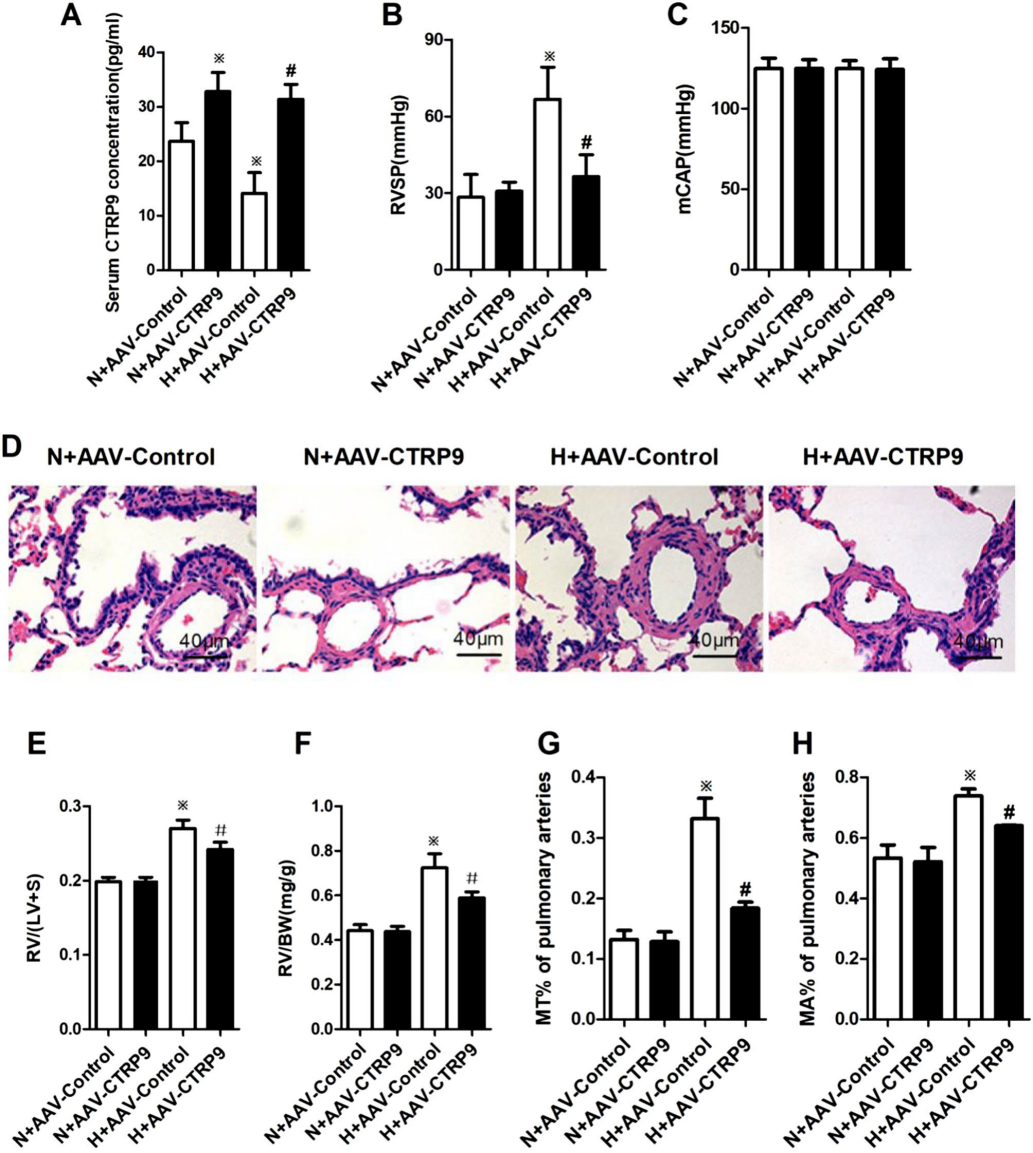
CTRP9 overexpression ameliorated HPH. (**A**) CTRP9 concentrations in rat serum are shown (n_N+AAV-Control_ = 4, n_N+AAV-CTRP9_ = 5, n_H+AAV-Control_ = 5, n_H+AAV-CTRP9_ = 6). (**B**, **C**) Quantitative data of cardiopulmonary hemodynamics, including right ventricular systolic pressure (RVSP; n_N+AAV-Control_ = 4, n_N+AAV-CTRP9_ = 7, n_H+AAV-Control_ = 6, n_H+AAV-CTRP9_ = 6) and mean carotid artery pressure (mCAP; n = 4), are presented. (**D**) Paraffin sections of rat lungs were assessed for pulmonary-arterial remodeling by staining (hematoxylin and eosin, × 400 magnification). Scale bar = 40 μm. (**E**, **F**) Indices of right ventricular remodeling are shown (E:n = 3; F: n_N+AAV-Control_ = 6, n_N+AAV-CTRP9_ = 7, n_H+AAV-Control_ = 6, n_H+AAV-CTRP9_ = 7). Data are the mean ± SEM. (**G**, **H**) Blinded quantitative analyses of MT% (n = 4) and MA% (n_N+AAV-Control_ = 4, n_N+AAV-CTRP9_ = 4, n_H+AAV-Control_ = 4, n_H+AAV-CTRP9_ = 5) of peripheral pulmonary arteries (30 vessels/3 sections per animal, 30–100 μm in diameter) were done using Image-Pro Plus. ^*※*^*P* < 0.05 vs. group N + AAV-Control; ^*#*^*P* < 0.05 vs. group H + AAV-Control. *N + AAV-Control* transfection of AAV-Control vectors maintained at normoxia for 4 weeks, *N + AAV-CTRP9* transfection of AAV-CTRP9 vectors maintained at normoxia for 4 weeks, *H + AAV-Control* transfection of AAV-Control vectors maintained at hypoxia for 4 weeks, *H + AAV-CTRP9* transfection of AAV-CTRP9 vectors maintained at hypoxia for 4 weeks.

To assess the effects of CTRP9 overexpression through gene transfer to rat lung tissues on plasma levels of CTRP9, the content of CTRP9 in rat serum was measured by enzyme-linked immunosorbent assays (ELISAs). The serum concentration of CTRP9 in group H + AAV-Control was decreased obviously as compared with that in group N + AAV-Control (*P* < 0.05). AAV-CTRP9 transfection increased the serum concentration of CTRP9 in contrast with that upon administration of AAV-Control under hypoxia (*P* < 0.05) (Fig. 2A). These data suggested that hypoxia induced down-regulation of CTRP9 expression in rat serum, and that transfer of AAV-CTRP9 vectors to lung tissues in rats could lead to CTRP9 overexpression and up-regulation of CTRP9 expression in rat serum under hypoxia.

After intermittent hypoxia for 28 days, RVSP (Fig. 2B), RVHI (Fig. 2E,F) and remodeling of small pulmonary arteries (Fig. 2D) presented as MT% (Fig. 2G) and MA% (Fig. 2H) in group H + AAV-Control were increased considerably compared with those in group N + AAV-Control. These results demonstrated that hypoxia induced RVSP enhancement, followed by remodeling of small pulmonary arteries and right ventricular hypertrophy, and that a HPH model in rats had been established. In contrast, the indicators noted above in group H + AAV-CTRP9 were reduced markedly compared with those in group H + AAV-Control. Neither hypoxia nor AAV transfection had an obvious impact on systolic blood pressure (Fig. 2C). Combined with alterations in serum levels of CTRP9 and survival of rats in different groups, these results suggested that CTRP9 could improve survival during hypoxia and diminish HPH.

### CTRP9 overexpression improved pulmonary endothelial function in HPH rats

To validate the mechanism by which CTRP9 alleviated HPH, the function of pulmonary arterioles was measured ex vivo.

**Figure 3:**
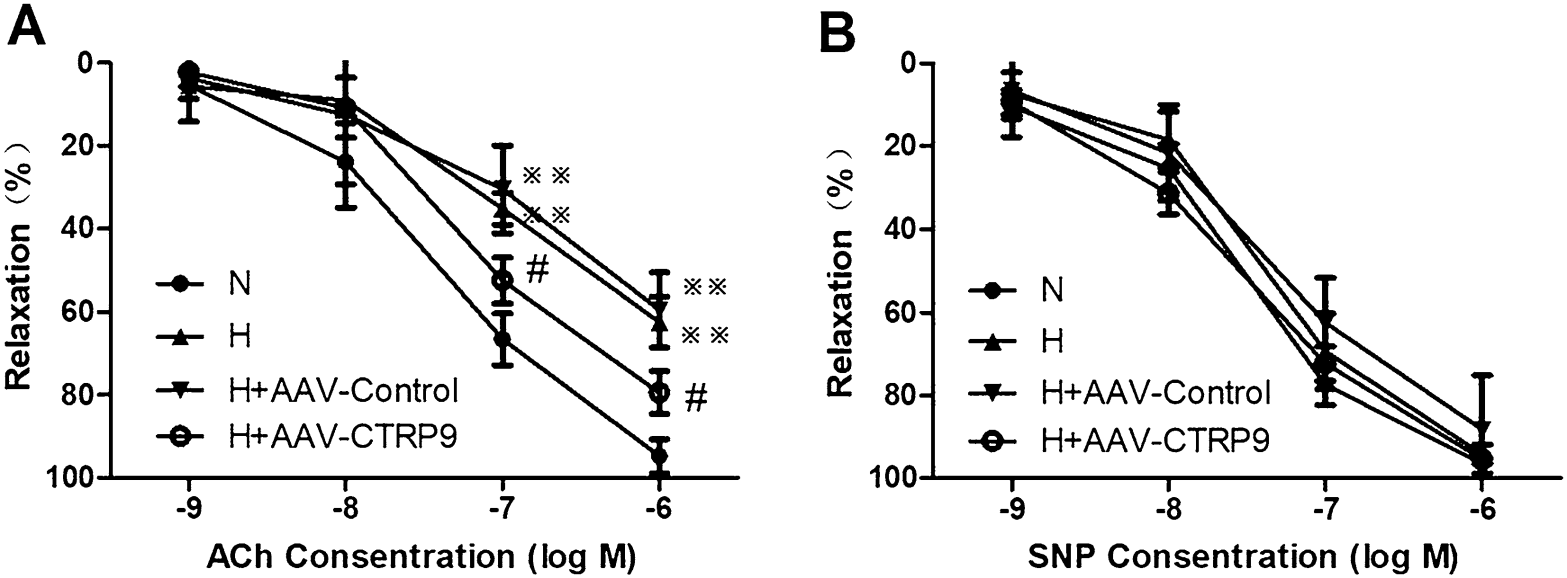
CTRP9 overexpression improved endothelial function in pulmonary arteries from HPH rats. (**A**) Dose–response curves for ACh-induced relaxation were obtained from pulmonary arteries of different groups (n = 6). Data are expressed as the percentage of the contraction to phenylephrine (PE). (**B**) Dose–response curves for SNP-induced relaxation were obtained from pulmonary arteries of different groups (n = 6). Data are expressed as the percentage of the contraction to PE. Data are the mean ± SEM. ^*※※*^*P* < 0.01 vs. group N; ^*#*^*P* < 0.05 vs. group H + AAV-Control. *N* normoxia for 4 weeks, *H4* hypoxia for 4 weeks, *H + AAV-Control* transfection of AAV-Control vectors maintained at hypoxia for 4 weeks, *H + AAV-CTRP9* transfection of AAV-CTRP9 vectors maintained at hypoxia for 4 weeks.

In pulmonary-artery rings from rats in groups N, H, H + AAV-Control or H + AAV-CTRP9, sodium nitroprusside (SNP; an endothelium-independent vasodilator) and acetylcholine (ACh; an endothelium-dependent vasodilator) stimulated vasodilation in a dose-dependent manner. Compared with rats from group N, vasodilator responses to SNP were not changed significantly in the pulmonary arteries from rats of group H (Fig. 3B). Importantly, as shown in Fig. 3A, the ability of ACh to cause dose-dependent vasorelaxation was reduced significantly in vascular segments from rats of group H than those from group N (*P* < 0.01), which suggested an impairment of pulmonary endothelial function in HPH. Compared with rats in group H, transfection with AAV-Control did not change the ACh-induced relaxation. Interestingly, the vasodilatation effect of ACh was improved markedly in the arterioles of rats from group H + AAV-CTRP9 compared with those from group N (*P* < 0.05) (Fig. 3A).

These results suggested that hypoxia attenuated the relaxing effects of ACh on pulmonary-artery rings, and that CTRP9 overexpression improved ACh-induced relaxation and endothelial function in HPH rats.

### CTRP9 overexpression promoted the balance of nitric oxide (NO) and ET-1 levels and regulated activation of endothelial nitric oxide synthase (eNOS) and extracellular signal-regulated kinase (ERK)1/2 differently

Pulmonary endothelial dysfunction has been shown to contribute to HPH development^12,13^, but how CTRP9 affects this pathologic process is not known. The molecules associated with this abnormal process in rat serum were quantified to help answer this important question.

**Figure 4:**
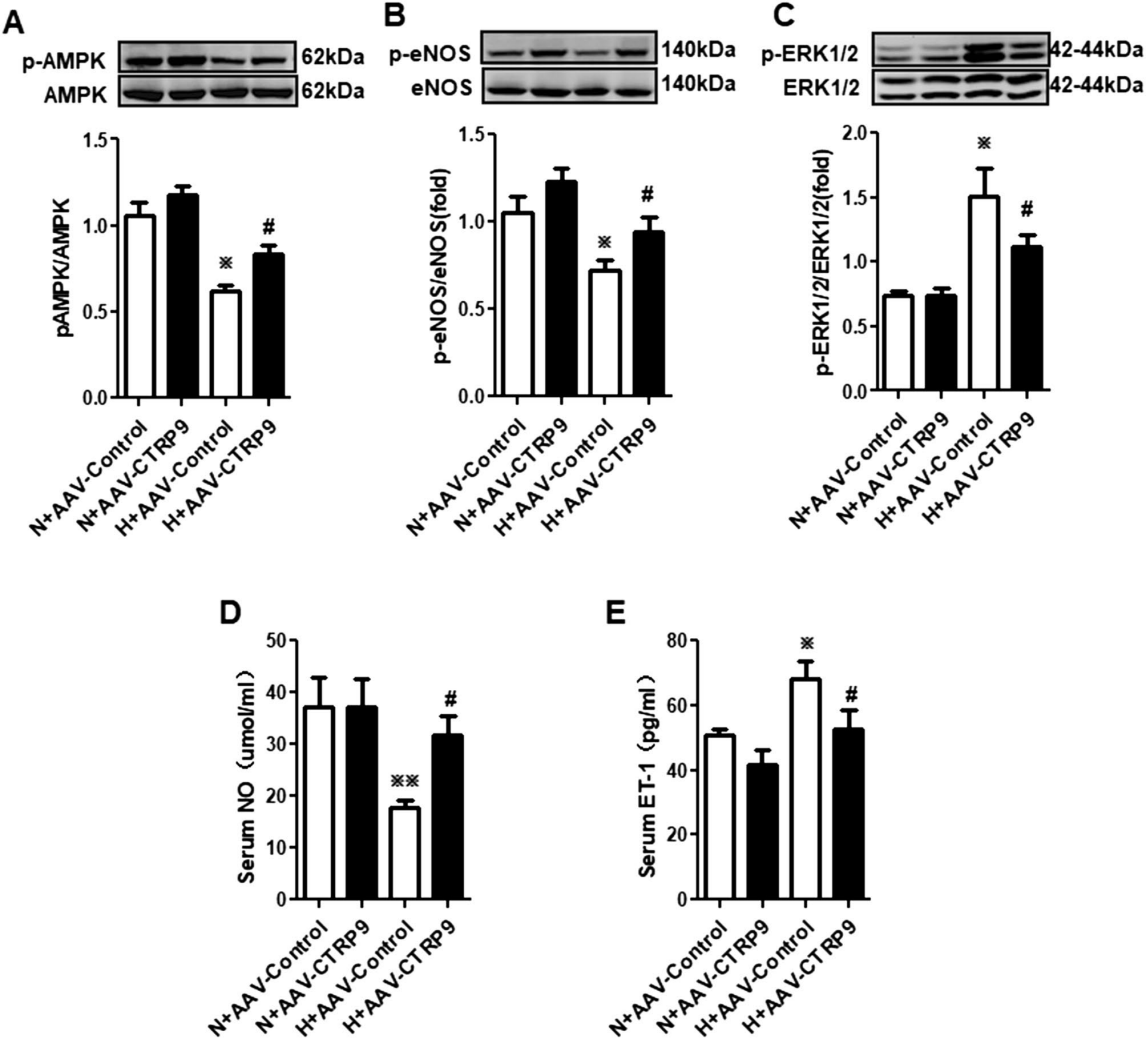
CTRP9 overexpression promoted phosphorylation of AMPK and eNOS and decreased ERK1/2 phosphorylation in isolated endothelial cells from the lungs of HPH rats. (**A**–**C**) Western blotting of expression of AMPK and p-AMPK (**A**, n = 3; blots are cropped from different gels), eNOS and p-eNOS (**B**, n = 3; blots are cropped from different gels), ERK1/2 and p-ERK1/2 (**C**, n = 3; blots are cropped from different gels) in endothelial cells from lungs of HPH rats using GAPDH as an internal control. The ratio of p-AMPK to AMPK, p-eNOS to eNOS, and p-ERK1/2 to ERK1/2 was calculated. (**D**, **E**) Levels of NO (n_N+AAV-Control_ = 3, n_N+AAV-CTRP9_ = 3, n_H+AAV-Control_ = 4, n_H+AAV-CTRP9_ = 3) and ET-1 (n_N+AAV-Control_ = 5, n_N+AAV-CTRP9_ = 5, n_H+AAV-Control_ = 4, n_H+AAV-CTRP9_ = 5) in rat serum were measured. Data are the mean ± SEM. ^*※*^*P* < 0.05, ^*※※*^*P* < 0.01 vs. group N + AAV-Control; ^*#*^*P* < 0.05 vs. group H + AAV-Control. *N + AAV-Control* transfection of AAV-Control vectors maintained at normoxia for 4 weeks, *N + AAV-CTRP9* transfection of AAV-CTRP9 vectors maintained at normoxia for 4 weeks, *H + AAV-Control* transfection of AAV-Control vectors maintained at hypoxia for 4 weeks, *H + AAV-CTRP9* transfection of AAV-CTRP9 vectors maintained at hypoxia for 4 weeks.

The content of NO and ET-1 (which reflect endothelial function) was measured (Fig. 4D,E). In the serum of rats in group H + AAV-Control, the NO concentration was reduced whereas the ET-1 level was enhanced (*P* < 0.05), compared with those in group N + AAV-Control. Interestingly, in group H + AAV-CTRP9, the NO concentration was increased (*P* < 0.01) and ET-1 concentration was reduced (*P* < 0.05) compared with those in group H + AAV-Control. These results suggested that hypoxia could disturb the production of NO and ET-1, and that CTRP9 could regulate such hypoxia-induced production. Hence, CTRP9 could ameliorate HPH by protecting the endothelium.

To investigate the potential mechanisms of action, expression of the upstream signaling molecules 5′-adenosine monophosphate-activated protein kinase (AMPK) and p-AMPK (phosphorylated at Thr^172^ of its α subunit), eNOS and p-eNOS (phosphorylated at Ser^1177^), ERK1/2 and p-ERK1/2 (phosphorylated at Thr^202^/Tyr^204^) was determined in isolated pulmonary microvascular endothelial cells (PMVECs). Total expression of AMPK, eNOS and ERK1/2 did not vary among the four groups (Fig. 4A–C). In group H + AAV-Control, expression of phosphorylated AMPK and eNOS was reduced, whereas that of phosphorylated ERK1/2 increased compared with those in group N + AAV-Control. In group H + AAV-CTRP9, phosphorylation levels of AMPK and eNOS was enhanced, and the phosphorylation level of ERK1/2 reduced compared with those in group H + AAV-Control. Alterations in expression of endothelial p-AMPK and p-eNOS corresponded to changes in NO expression, and alterations in expression of p-ERK1/2 corresponded with alterations in ET-1 expression.

These results suggested that CTRP9 contributed to NO production and restrained ET-1 production during hypoxia. We speculated that, during hypoxia, CTRP9 contributed to eNOS phosphorylation to produce NO, and restrained ERK1/2 phosphorylation to reduce ET-1 expression.

### AMPK mediated CTRP9-induced NO production and reduced ET-1 production by activating eNOS and inactivating ERK1/2

Furthermore, we wished to ascertain if AMPK: (1) mediated the effect of CTRP9 on endothelial function; (2) was a key regulator for eNOS phosphorylation and ERK1/2 phosphorylation. Hence, the concentration of NO and ET-1, and expression of AMPK/p-AMPK, eNOS/p-eNOS, and ERK1/2/p-ERK1/2, in cultured PMVECs were measured.

**Figure 5:**
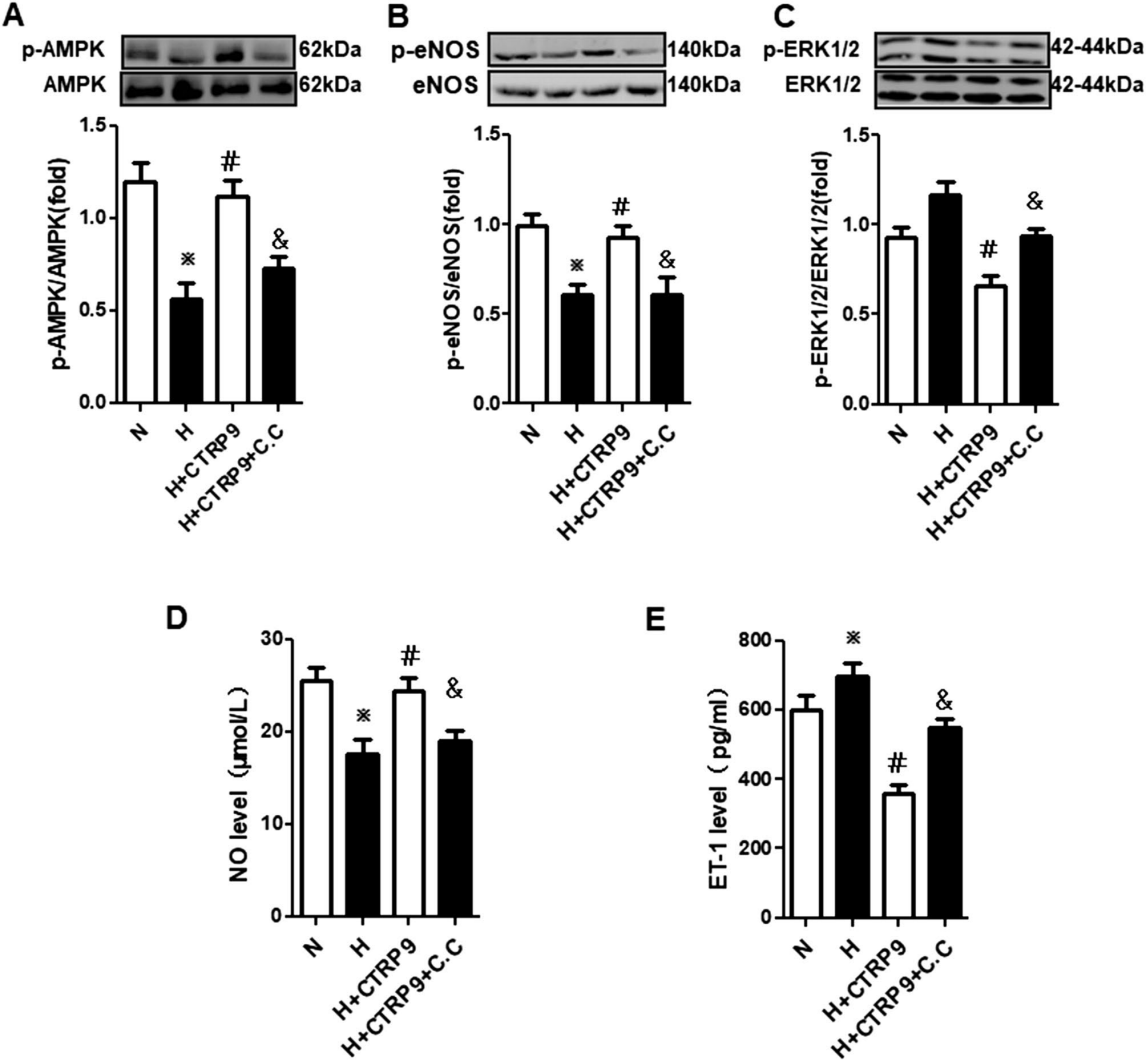
AMPK mediated CTRP9-induced NO production and reduced ET-1 production by activating eNOS and inactivating ERK1/2. (**A**–**C**) Western blotting of expression of AMPK and p-AMPK (**A**, n = 3; blots were cropped from different parts of the same gel), eNOS and p-eNOS (**B**, n = 3; blots are cropped from different gels), ERK1/2 and p-ERK1/2 (**C**, n = 3; blots cropped from different gels) in rat PMVECs. (**D**, **E**) Levels of NO (n_N+AAV-Control_ = 3, n_N+AAV-CTRP9_ = 3, n_H+AAV-Control_ = 5, n_H+AAV-CTRP9_ = 4) and ET-1 (n_N+AAV-Control_ = 5, n_N+AAV-CTRP9_ = 5, n_H+AAV-Control_ = 5, n_H+AAV-CTRP9_ = 6) in PMVECs culture medium. Data are the mean ± SEM. ^※^*P* < 0.05 vs. group N; ^*#*^*P* < 0.05 vs. group H; ^*&*^*P* < 0.05 vs. group H + CTRP9. *H* hypoxia, *N* normoxia, *C.C* compound C.

NO production, the AMPK phosphorylation level, and eNOS phosphorylation level were reduced, and ET-1 production (*P* < 0.05) and the ERK1/2 phosphorylation level induced, in group H compared with group N (Fig. 5A–E). Compared with group H, NO production, the AMPK phosphorylation level and eNOS phosphorylation level were increased, and ET-1 production and the ERK1/2 phosphorylation level attenuated in group H + CTRP9 (*P* < 0.05). In contrast with group H + CTRP9, pretreatment with compound C before hypoxia and CTRP9 treatment inhibited the effect of CTRP9 on p-AMPK/p-eNOS/NO and p-ERK1/2/ET-1 signaling pathways in PMVECs. These results suggested that CTRP9 regulated production of the vasoactive substances NO and ET-1 through AMPK-mediated phosphorylation of eNOS and ERK1/2 in PMVECs.

**Figure 6:**
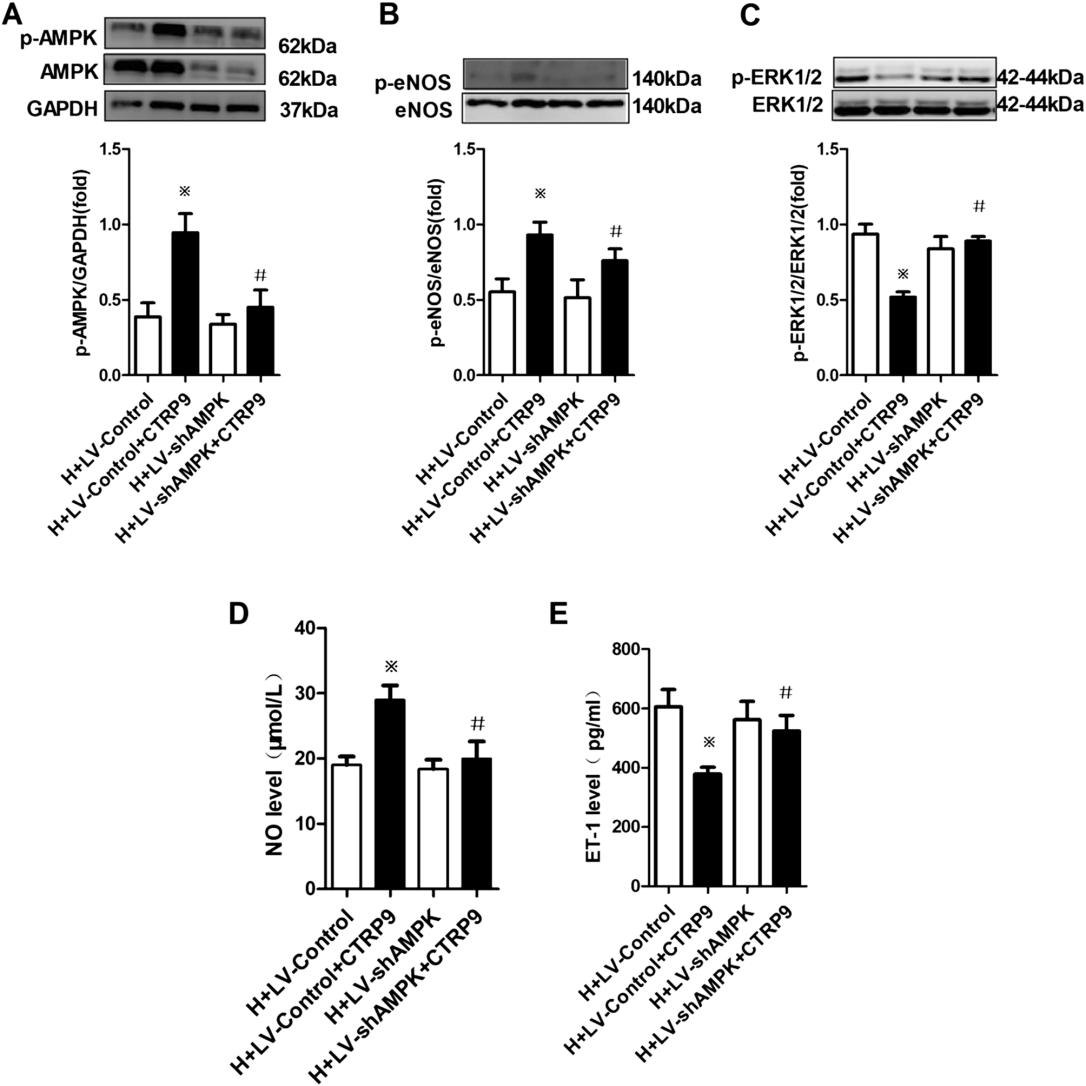
Knockdown of AMPK expression inhibited CTRP9-induced alterations in expression of p-AMPK, p-eNOS, p-ERK1/2, NO and ET-1. (**A**–**C**) Western blotting of expression of AMPK and p-AMPK (**A**, n = 3; blots were cropped from different parts of the same gel), eNOS and p-eNOS (**B**, n = 3; blots were cropped from different gels), ERK1/2 and p-ERK1/2 (**C**, n = 3; blots were cropped from different gels) in rat PMVECs. The protein ratio of p-AMPK to AMPK, p-eNOS to eNOS, and p-ERK1/2 to ERK1/2 was calculated. (**D**, **E**) Levels of NO (n = 3) and ET-1 (n = 4) in PMVECs culture medium were examined. Data are the mean ± SEM. ^※^*P* < 0.05 vs. H + LV-Control group; ^*#*^*P* < 0.05 vs. H + LV-Control + CTRP9 group. *H* hypoxia, *LV-Control* lentivirus control, *LV-shAMPK* lentivirus with AMPK interruption.

Additional experiments were conducted using lentivirus to suppress AMPK expression to obtain direct evidence supporting the causative role of AMPK activation in CTRP9-based regulation of production of NO and ET-1. Twenty-four hours after transfection with LV-GFP-shAMPK or LV-GFP-con, PMVECs were incubated with or without CTRP9 (5 μg/mL) for 48 h under hypoxia. Western blotting showed that LV-GFP-shAMPK suppressed AMPK expression accompanied by suppression of AMPK phosphorylation (Fig. 6A). This action impeded CTRP9-induced increased the eNOS phosphorylation level and NO production, and reduced the ERK1/2 phosphorylation level and ET-1 production under hypoxia (Fig. 6B–E). These results showed the core responsibility of AMPK in CTRP9-induced endothelial protection.

## Discussion

Initially, we found a correlation between decreased serum concentrations of CTRP9 and HPH development in rats. This finding is consistent with that of Li et al. in PH patients and HPH rats, and implies that CTRP9 might protect against PH^14,15^. In further studies, we overexpressed CTRP9 in HPH rats and demonstrated that CTRP9 could ameliorate alterations in pulmonary hemodynamics (as shown by RVSP), pulmonary vascular remodeling (as shown by MT% and MA%), right ventricular hypertrophy (as shown by RVHI) in HPH rats and aid survival. Then, we demonstrated that such amelioration resulted from CTRP9-induced improvement in pulmonary endothelial function. Specifically, CTRP9 restored the balance between the vasoconstrictor ET-1 and vasodilator NO secreted from the pulmonary endothelium. The underlying mechanism involved AMPK-mediated up-regulation of p-eNOS expression, which contributed to NO production, and AMPK-mediated down-regulation of p-ERK1/2 expression, which restrained ET-1 production. These novel results could lead to new therapeutic strategies for HPH (“Supplementary Information”).

CTRP9 has multiple beneficial effects against disorders of the cardiovascular system or metabolism^8,9^. CTRP9 can relax arteries by inducing NO production, and the underlying mechanism is associated with activation of the AMPK/protein kinase B (AKT)/eNOS signaling pathway^10^. In addition, CTRP9 can attenuate proliferation of vascular smooth muscle cells and prevent vascular restenosis after vascular injury^11^.

Although CTRP9 has been feted ubiquitously with regard to cardiovascular disorders because of its beneficial effects, the influence of CTRP9 on PH has not been studied in depth. Only Li et al., have described the association of CTRP9 and PH. Their first study reported that the serum level of CTRP9 in PH patients was low, and that CTRP9 overexpression induced by plasmid cloning (pc)DNA-CTRP9 could reduce the pulmonary arterial pressure in a PH model induced by an inferior-vena-cava shunt^14^ Their next study showed that CTRP9 could suppress the proliferation and migration of pulmonary arterial smooth muscle cells via suppression of a transforming growth factor-1/ERK1/2 signaling pathway during hypoxia^15^. Thus, we wished to elucidate how CTRP9 affects pulmonary endothelial functions in HPH.

Hypoxia-induced injury/dysfunction of pulmonary endothelial cells leads to HPH^16^. A growing body of literature has shown that AMPK, a crucial regulator of cellular-energy metabolism^17^ and modulator of vascular reactivity^18,19^, protects the endothelium and is involved in vasodilation. Using pulmonary-artery rings, we found that CTRP9 overexpression improved ACh-induced endothelium-dependent pulmonary-artery relaxation. the AMPK phosphorylation level was decreased in the lung endothelial cells of HPH rats, and CTRP9 overexpression could increase AMPK phosphorylation level and ameliorate HPH. The results of in vivo, ex vivo and in vitro experiments suggested that AMPK-mediated endothelial protection has a core role in the effects of CTRP9.

Endothelial dysfunction involves the expression and secretion of vasoactive substances such as NO and ET-1. NO can be synthesized by eNOS in endothelial cells^4^. NO has functions in vasodilation^20^ and curbing of the proliferation of vascular smooth muscle cells^21^. ET-1 is derived from preproendothelin, which is generated and secreted from endothelial cells^22^, and which has roles in vasoconstriction and proliferation of vascular smooth muscle cells^23^. Up-regulation of an ET-1 signaling pathway^5,24,25^ and down-regulation of a NO pathway^26^ have been demonstrated to be involved in HPH development. Moreover, in clinical practice, the efficacy of compounds developed on the basis of the abnormal signaling pathways of NO^27^ and ET-1^28^ have provided direct evidence of their roles in HPH development. AMPK has been reported to activate eNOS by phosphorylating eNOS at Ser^1177^ and inactivate ERK1/2 by reducing ERK1/2 phosphorylation level at Thr^202^/Tyr^204^ in the systemic circulation^29^. Moreover, Zheng et al.^10^ reported that CTRP9 can dilate the aorta by activating an AMPK/eNOS/NO signaling pathway. Thus, we speculated that during hypoxia, CTRP9 might contribute to eNOS phosphorylation to produce NO, and restrain ERK1/2 phosphorylation to reduce ET-1 expression. We hypothesized that AMPK is a key regulator for the phosphorylation of eNOS and ERK1/2.

We observed that corresponding to a decrease in the AMPK phosphorylation level in the lung endothelial cells of HPH rats, the eNOS phosphorylation level and NO production were decreased, and that the ERK1/2 phosphorylation level and ET-1 production were increased. After overexpressing CTRP9 using AAV-CTRP9, the AMPK phosphorylation level was increased, and corresponded with an increase in the eNOS phosphorylation level and NO production, and a decrease in the ERK1/2 phosphorylation level and ET-1 production. We undertook additional experiments to identify the relationship between AMPK and eNOS/NO and ERK1/2/ET-1. Through addition of compound C (an inhibitor of AMPK phosphorylation) to cell-culture media before CTRP9 treatment, or knockdown of AMPK expression in PMVECs, we showed that CTRP9 could enhance the eNOS phosphorylation level and NO production, and reduce the ERK1/2 phosphorylation and level ET-1 production, in an AMPK-dependent fashion under hypoxia. These data are in accordance with our results for pulmonary-artery rings. Taken together, these results clearly showed that AMPK was the principal kinase responsible for CTRP9-induced eNOS phosphorylation and NO production, CTRP9-based reduction of ERK1/2 phosphorylation and ET-1 production and, subsequently, induction of relaxation of pulmonary arteries.

We found that CTRP9 could suppress AMPK-dependent ERK1/2 phosphorylation and ET-1 production. Uemura et al. reported that CTRP9 could attenuate neointimal formation via a cyclic adenosine monophosphate/protein A pathway, which might be associated with suppressing ERK1/2 phosphorylation^11^. Li et al.^15^ found that CTRP9 could decrease ET-1 production via a phosphatidylinositol 3-kinase/AKT pathway in endothelial cells during normoxia. This difference in results could have arisen from the different pathologic state and tissues used in their study and the present study.

With regard to the contradiction between the short half-life of CTRP9 and longer period of construction of an HPH model in rats, CTRP9 administration (via tail-vein injection) should be done once per day at least. With increasing time, the tail vein will suffer stenosis and occlusion and, consequently, the absorption effects will be disturbed.

AAV has been used widely in gene therapy because it is efficacious, safe, can be used in different tissues, and has fewer side effects, inflammatory reactions, and immune reactions than those using other vectors. Moreover, AAV can express the target gene continually without DNA integration into the host chromosome^30^. Gene transfection to the pulmonary vascular bed through intra-tracheal instillation has been demonstrated to be effective, safe and feasible^31^. Therefore, we selected AAV as a vector through intra-tracheal instillation to overexpress CTRP9 in rats. Before the present study, we tested the efficiency of AAV6 transfer to lung tissues through intra-tracheal instillation, as well as the onset and persistence of gene expression (data not shown).

Traditional mechanism-based pharmacologic drugs focus mainly on one of three main pathogenic signaling pathways of PH. CTRP9 can focus on NO and ET-1 pathways, which could strengthen efficacy and reduce treatment costs. AAV was safe and could express the target gene consistently in vivo. These features will avoid frequent administration of drugs and improve patient compliance with drug therapy. Moreover, intra-tracheal instillation lays the foundation for aerosol nebulization, which would enable elicitation of the effects of CTRP9 in the pulmonary circulation specifically. However, we did not examine whether CTRP9 overexpression could rescue HPH in rats, which warrants our further study in the future.

## Conclusions

CTRP9 ameliorates hypoxia-induced pulmonary hypertension in rats. This is achieved through protection of the functions of pulmonary vascular endothelial cells. CTRP9 contributes to AMPK phosphorylation and restores the balance between eNOS/NO and ERK1/2/ET-1. These results aid understanding of the molecular pathogenesis of HPH and, using CTRP9 as a novel therapy, prevent HPH development.

## Materials and methods

### Ethical approval of the study protocol

The study protocol was approved by the Ethics Committee of The Fourth Military Medical University (Xi’an, China). Animals were bred and maintained in accordance with the guidelines of the Animal Care and Use Committee of The Fourth Military Medical University. The study was carried out in compliance with ARRIVE guidelines.

### Animal handling and experimental design

Male Sprague-Dawley rats (180–220 g) were provided by the Experimental Animal Center of the Fourth Military Medical University.

First, rats were divided randomly into three groups: (1) normoxia for 4 weeks (group N); (2) hypoxia for 2 weeks (group H2); (3) hypoxia for 4 weeks (group H4), n = 6.

To explore the impact of CTRP9 on HPH development, rats were divided randomly into four groups according to transfection with: (1) AAV-control vectors and maintained at normoxia for 4 weeks (group N + AAV-Control); (2) AAV-CTRP9 vectors and maintained at normoxia for 4 weeks (group N + AAV-CTRP9); (3) AAV-Control vectors maintained at hypoxia for 4 weeks (group H + AAV-Control); (4) AAV-CTRP9 vectors maintained at hypoxia for 4 weeks (group H + AAV-CTRP9). Rats in group N were maintained in a normal condition for 4 weeks.

Rats in groups H2 or H4 were maintained intermittently in a hypobaric hypoxia chamber, with fractional inspired oxygen of 10% at 8 h/day for 2 weeks and 4 weeks, respectively. Rats in groups N + AAV-Control, N + AAV-CTRP9, H + AAV-Control or H + AAV-CTRP9, before being moved to normoxia or hypoxia conditions for 4 weeks, respectively, were pretreated by transfection with AAV-Control or AAV-CTRP9 and housed under normoxia for 3 weeks in accordance with the preliminary experiment. All animals were kept in individual ventilated cages placed in a temperature-controlled room with a 12-h light–dark cycle.

### Construction of AAV vectors and mediated gene transfer to rat lungs

AAV is an efficient and safe vector for gene transfer *in vivo*^32^, and serotype 6 is specific for lung tissue^33^. Thus, the fragment comprising the entire protein-coding region of rat CTRP9 was cloned into the AAV vector (pHBAAV-CMV-MCS-ZsGreen), which was driven by a cytomegalovirus promoter and harbored a GFP region. Scrambled AAV vectors were utilized as a negative control. Recombinant AAV was plaque-purified, and the titer measured by a plaque assay on cells in culture at 1 × 10^11^ viral genomes. After purification, recombinant AAV was suspended in phosphate-buffered saline (pH 7.4) and maintained at − 80 °C until use. All procedures were undertaken by Hanheng Biotechnology (Shanghai, China).

As described previously^34^, AAV vectors can be transferred into the pulmonary vascular bed through intra-tracheal instillation. Briefly, male rats were anesthetized (isoflurane 1–2%) and placed supine on a surgical table. Using a sterile method, the trachea was approached via a midline incision in the neck and isolated by blunt dissection. A polyethylene tube (PE-240; 1.67-mm ID) was inserted into the trachea with a needle. A microliter syringe was attached to this polyethylene tube. The latter was parallel to the trachea wall and extended to the bronchi. A virus solution (50 μL) was instilled by injection into right or left bronchi with the rat in the right or left lateral recumbent position, respectively. When drawing the virus solution into the syringe before administration, an additional volume of air (0.2 mL) was drawn into the syringe to force all of the virus solution from the hub of the syringe and needle into the trachea. After completion of procedures, rats were maintained and monitored in sternal recumbency in a quiet cage with their head and thorax elevated slightly until ambulation.

### Hemodynamic measurements and tissue preparation

Hemodynamic evaluation was undertaken after the final hypoxic and hypobaric treatment. During the procedure, the temperature, respiration, and heart rate remained stable as verified by continuous monitoring. Briefly, rats were anesthetized with 3% pentobarbital sodium. An incision was made to expose the right external jugular vein. Subsequently, a micro-incision in this vein was made to insert a polyethylene catheter with PE-240 tubing (1.67-mm ID) connected to a pressure transducer. The catheter passed through the right atrium and advanced into the right ventricle. The RVSP was measured to estimate the pulmonary arterial pressure^35^. Then, another polyethylene catheter (PE-240 tubing; 1.67-mm ID) was inserted into the left carotid artery. It was connected to a pressure transducer to measure systemic arterial pressure. Pressure was recorded on a PowerLab system (AD Instruments, Castle Hill, New South Wales, Australia). After measurements, blood was collected from the arterial catheter for subsequent separation of plasma. Then, rats were exsanguinated, and their hearts and lungs were removed. The right lower lobes of lungs were dissected and blotted, followed by embedding with 4% paraformaldehyde to prepare serial slices.

### Measurement of right ventricular mass

To determine right ventricular hypertrophy, the free wall of the right ventricle was dissected from the left ventricle and septum, and each portion weighed. The RVHI was calculated using the following equation:

$$ {\text{RVHI}} = {{{\text{RV}}} \mathord{\left/ {\vphantom {{{\text{RV}}} {\left( {{\text{LV}} + {\text{S}}} \right)}}} \right. \kern-\nulldelimiterspace} {\left( {{\text{LV}} + {\text{S}}} \right)}}\;{\text{and}}\;{\text{RV/BW}} $$

where RV is the right ventricle, LV is the left ventricle, S is the septum and BW is body weight.

### Vascular morphometry

To assess morphologic changes in pulmonary arteries, the right-upper lung lobes were immersed in paraformaldehyde buffer (40 ml/L) for 24 h to create paraffin sections, and these paraffin sections were stained with hematoxylin and eosin. Peripheral pulmonary arteries (30–100 μm in diameter, 10 vessels per section), running along the terminal and respiratory bronchioles as well as alveolar duct were captured randomly using a digital photomicrograph (Leica, Heidelberg, Germany). For peripheral pulmonary arteries, the MT% was calculated using the following equation:

$$ {\text{MT}}\% = {1}00 \times {{\left[ {{\text{thickness}}\;{\text{of}}\;{\text{medial}}\;{\text{layer}}} \right]} \mathord{\left/ {\vphantom {{\left[ {{\text{thickness}}\;{\text{of}}\;{\text{medial}}\;{\text{layer}}} \right]} {\left[ {{\text{vessel}}\;{\text{semi - diameter}}} \right]}}} \right. \kern-\nulldelimiterspace} {\left[ {{\text{vessel}}\;{\text{semi - diameter}}} \right]}} $$

For peripheral pulmonary arteries, the MA% was calculated using the following equation:

$$ {\text{MA}}\% = {1}00 \times {{\left[ {{\text{cross - sectional}}\;{\text{area}}\;{\text{of}}\;{\text{the}}\;{\text{medial}}\;{\text{layer}}} \right]} \mathord{\left/ {\vphantom {{\left[ {{\text{cross - sectional}}\;{\text{area}}\;{\text{of}}\;{\text{the}}\;{\text{medial}}\;{\text{layer}}} \right]} {\left[ {{\text{total}}\;{\text{cross - sectional}}\;{\text{area}}\;{\text{of}}\;{\text{the}}\;{\text{vessel}}} \right]}}} \right. \kern-\nulldelimiterspace} {\left[ {{\text{total}}\;{\text{cross - sectional}}\;{\text{area}}\;{\text{of}}\;{\text{the}}\;{\text{vessel}}} \right]}} $$

These two parameters of peripheral pulmonary arteries were analyzed in a blinded way using Image-Pro Plus v6.0 (Media Cybernetics, Rockville, MD, USA).

### Measurement of isometric tension

For measurement of isometric tension, the methods of anesthesia and euthanasia were as described above^36^. Pulmonary arteries isolated from rats were cut into 3-mm rings and placed in oxygen-saturated Krebs solution (pH 7.4) (Krebs solution had the following composition (in mmol/L): NaCl, 118; glucose, 11; KCl, 4.7; CaCl_2_, 2.5; NaHCO_3_, 25; KH_2_PO_4_, 1.2; and MgCl_2_, 1.2). Each ring was suspended with a force transducer at a preload of 1 g to record changes via a data-acquisition system (BL-420; Chengdu Taimeng, Chengdu, China). Rings were equilibrated for 1 h with three washouts in normal Krebs solution (pH 7.4, 37 °C) bubbled with a gas mixture of 95% O_2_ and 5% CO_2_ continuously. After equilibration, phenylephrine (PE; l μmol/L) was added to the bath. Once stable contraction had been reached, a concentration–response curve to ACh and SNP was constructed.

### Isolation of PMVECs

PMVECs were isolated as described previously^37,38^ After anesthesia and euthanasia as described above, the outer ~ 4 mm of peripheral lung tissue was resected from rats of different groups. Then, digestion was carried out by submerging the tissue in type-II collagenase (Worthington Biochemical, NJ, USA) for 20 min at 37 °C in an atmosphere of 5% CO_2_ and 90% humidity. Next, the digested lung fragments were transferred to a culture dish containing endothelial cell-specific culture medium (M-200; Sigma–Aldrich, Saint Louis, MO, USA), supplemented with 20% fetal bovine serum (FBS) and 1% low serum growth supplement (Gibco, Billings, MT, USA) and 1% penicillin/streptomycin (Thermo Fisher, Waltham, MA, USA), minced with sterile scissors (~ 100 times), and the tissue homogenate was filtered through a 70-μm cell strainer. Then, the filtrate was passed through a 20-μm strainer. PMVECs were purified from the cell suspension using positive selection with an Anti-CD31 (PECAM-1) Antibody MicroBeads Kit (Magnetic Cell Sorting; Miltenyi Biotec, Bergisch Gladbach, Germany). The endothelial phenotype was confirmed by fluorescence microscopy using anti-mouse CD31 antibody.

### Cell culture

Primary PMVECs from rats were prepared through a tissue-explant method^39,40^. Briefly, after anesthesia and euthanasia as described above, the lung fringe (< 1 mm) from rats was dissected and cut into pieces (~ 0.5 mm^3^) in FBS. Then, the pieces were placed evenly at the bottom of the cell culture flask and plated for 3 h without media at 37 °C in a humidified atmosphere of 5% CO_2_ and air, which allowed the pieces to attach to the culture flasks. After attachment, the pieces were cultured in endothelial cell media (M-200; Sigma–Aldrich) with 20% FBS and 1% low serum growth supplement (Gibco). After culture for 48 h, the pieces were centered on new-grown cells, and then removed to avoid mixing with other cell types. PMVECs within 2–4 passages were chosen. PMVECs were synchronized by endothelial basal media containing 5% FBS for 12 h before experimentation.

PMVECs grown to sub-confluence were exposed to normoxia (group N), hypoxia (group H), hypoxia with CTPR9 (final concentration: 5 μg/mL) treatment (group H + C9), and compound-C (Selleckchem, Houston, TX, USA) treatment 30 min before treatment with hypoxia and CTRP9 (group H + C9 + CC) for 48 h, respectively. For exposure to hypoxia, PMVECs were placed in a hypoxia incubator (Thermo Fisher) at 37 °C in a humidified atmosphere of 5% O_2_/5% CO_2_/90% N_2_. PMVECs in culture media were harvested to measure the level of NO and ET-1. Cells samples were used to detect the protein level of AMPK/p-AMPK, eNOS)/p-eNOS and ERK1/2/p-ERK1/2.

### Construction and transfection of lentivirus

To investigate the influence of AMPK on CTRP9-induced NO and CTRP9-induced reduction of ET-1 production, short hairpin (sh)RNA (shAMPK, 5′-TAAAGTAGCTGTGAAGATA-3′) targeting a specific region of AMPK mRNA in rats, and a scrambled negative control (sh-con, 5′-TTCTCCGAACGTGTCACGT-3′), were cloned into the pGCSIL-004 vector (Genechem, Shanghai, China). The lentivirus was synthesized by Genechem.Lentivirus with scrambled shRNA was used as a negative control (LV-GFP-sh-con). In accordance with the results of the preliminary experiment, the multiplicity of infection of PMVECs for lentivirus was 20:1. PMVECs were seeded in a six-well dish with 2-mL cell suspensions (6 × 10^4^/mL) for 24 h. LV-GFP-shAMPK or LV-GFP-sh-con was added to the medium to enable treatment for 12 h. Then, the media with lentivirus were replaced by endothelial basal media containing 5% FBS for an additional 12 h to aid synchronization of PMVECs. Then, PMVECs were incubated in the absence or presence of CTRP9 (5 μg/mL) for 48 h under hypoxia. PMVECs in culture media were harvested to measure expression of NO and ET-1. Cell lysates were used to measure the protein level of AMPK/p-AMPK, eNOS/p-eNOS and ERK1/2/p-ERK1/2.

### Western blotting

Cell samples were collected and lysed in RIPA lysis buffer (Beyotime, Beijing, China). Lysates were centrifuged at 14,000 × *g* for 20 min at 4 °C, and the supernatant used. The protein concentration was determined using a bicinchoninic acid assay kit (Beyotime). Protein suspensions from different groups containing equal amounts of proteins were separated by sodium dodecyl sulfate–polyacrylamide gel electrophoresis and transferred to polyvinylidene fluoride (PVDF) membranes (Millipore, Billerica, MA, USA). Then, PVDF membranes were blocked with 5% non-fat milk solution and western-blotted overnight with appropriate primary antibodies: anti-p-AMPK (Thr^172^), anti-AMPK, anti-p-ERK1/2 and anti-ERK1/2 at 1:1000 dilution (Cell Signaling Technology, Danvers, MA, USA); anti-p-eNOS (Ser^1177^) and anti-eNOS at 1:500 dilution (BD Biosciences, San Jose, CA, USA); anti-glyceraldehyde 3-phosphate dehydrogenase at 1:2000 dilution (CW Biotech, Beijing, China). Secondary to western blotting, PVDF membranes were incubated with the corresponding horseradish peroxide-conjugated antibody solution for 1 h. The blot was probed through an enhanced chemiluminescence reagent (Millipore). Blot densities were analyzed using ImageJ (National Institutes of Health, Bethesda, MD, USA).

### Quantification of NO release in rat serum and endothelial-cell medium

Total NO production in the culture medium or serum involved measurement of the concentration of nitrite (a stable metabolite of NO) with a modified Griess reaction as reported previously^41^. Values are expressed as μmol/mL.

### Determination of the concentration of CTRP9 and ET-1 in serum or cell-culture medium

Total CTRP9 concentration in serum, as well as levels of ET-1 in the culture medium or serum, were measured with a sensitive ELISA kit (Shanghai Westang BioTech, Shanghai, China) according to manufacturer instructions. Values are expressed in pg/mL.

### Statistical analyses

Data are the mean** ± **SD. Differences were analyzed by one-way ANOVA if three or more groups were compared. *P* < 0.05 was considered significant. All statistical tests were undertaken with SPSS v16.0 (IBM, Armonk, NY, USA).

## Supplementary Information

Supplementary Legends.

Supplementary Figures.
