# Supplementary material for: C1q/TNF-related protein-9 ameliorates hypoxia-induced pulmonary hypertension by regulating secretion of endothelin-1 and nitric oxide mediated by AMPK in rats
Source: Sci Rep. 2021 May 31;11:11372. doi: 10.1038/s41598-021-90779-2 (PMC8166879; doi:10.1038/s41598-021-90779-2)
Supplement: Supplementary file 1 — Supplementary Legends. [file 41598_2021_90779_MOESM1_ESM.docx]

**Supplementary legend**

**Figure 1.** CTRP9 overexpression promoted phosphorylation of AMPK and eNOS and decreased ERK1/2 phosphorylation in isolated endothelial cells from lungs of HPH rats. A-C: Western blotting of expression of AMPK and p-AMPK (A，n=3; the blots cropped from different gels), eNOS and p-eNOS (B, n=3; the blots cropped from different gels), ERK1/2 and p-ERK1/2 (C, n=3; the blots cropped from different gels) in endothelial cells from lungs of HPH rats using GAPDH as an internal control. N+AAV-Control: transfection of AAV-Control vectors maintained at normoxia for 4 weeks; N+AAV-CTRP9: transfection of AAV-CTRP9 vectors maintained at normoxia for 4 weeks; H+AAV-Control: transfection of AAV-Control vectors maintained at hypoxia for 4 weeks; H+AAV-CTRP9: transfection of AAV-CTRP9 vectors maintained at hypoxia for 4 weeks.

**Figure 2.** AMPK mediated CTRP9 induced-NO production and reduced ET-1 production by activating eNOS and inactivating ERK1/2. A-C: Western blotting of expression of AMPK and p-AMPK (A, n=3; the blots cropped from different part of the same gel), eNOS and p-eNOS (B,n=3; the blots cropped from different gels), ERK1/2 and p-ERK1/2 (C, n=3; the blots cropped from different gels) in rat PMVECs. H: hypoxia; N: normoxia; C.C: compound C.

**Figure 3.** Knockdown of AMPK expression inhibited CTRP9-induced alterations in expression of p-AMPK, p-eNOS, p-ERK1/2, NO and ET-1. A-C: Western blotting of expression of AMPK and p-AMPK (A, n=3, the blots cropped from different part of the same gel), eNOS and p-eNOS (B, n=3, the blots cropped from different gels), ERK1/2 and p-ERK1/2 (C,n=3, the blots cropped from different gels) in rat PMVECs. The protein ratio of p-AMPK to AMPK, p-eNOS to eNOS, p-ERK1/2 to ERK1/2 was calculated. H: hypoxia; LV-Control: lentivirus control; LV-shAMPK: lentivirus with AMPK interruption.

**Figure 4.** The expression level of GFP in lung tissues from rat with AAV-6 intra-tracheal instillation were detected. there were no differences among the four groups in GFP expression, which suggested a same transfection effect of AAV-control or AAV-CTRP9 to lung tissues of rats in hypoxic or normoxic condition.

**Figure 5.** Assessment of the inhibitory effects of lentiviruses on AMPK with regard to expression of protein was done by western blotting. As presented in supplementary Figure 5, in contrast to sh-control or media-control, sh-AMPK lentivirus infection reduced the AMPK protein expression and can be used in further experiments to investigate the effects of CTRP 9 on vasoactive substance in PMVECs.
