# Supplementary material for: C1q/TNF-related protein-9 ameliorates hypoxia-induced pulmonary hypertension by regulating secretion of endothelin-1 and nitric oxide mediated by AMPK in rats
Source: Sci Rep. 2021 May 31;11:11372. doi: 10.1038/s41598-021-90779-2 (PMC8166879; doi:10.1038/s41598-021-90779-2)

Article

C1q/TNF-related protein-9 ameliorates hypoxia-induced pulmonary hypertension by regulating secretion of endothelin-1 and nitric oxide mediated by AMPK in rats

Qiaoyan Jin[1*], Hui Su[2*], Rui Yang[1*], Yanzhen Tan[3], Buying Li[3], Wei Yi[3], Qianqian Dong[4], Haifeng Zhang[4], Wenjuan Xing[5#], Xin Sun[1#]

[1]Department of Pediatrics, Xijing Hospital, Fourth Military Medical University, Xi'an, 710032, China

[2]Department of Geriatrics, Xijing Hospital, Fourth Military Medical University, Xi'an, 710032, China

[3]Department of Cardiovascular Surgery, Xijing Hospital, Fourth Military Medical University, Xi'an, 710032, China

[4]Teaching Experiment Center, Fourth Military Medical University, Xi'an, 710032, China

[5]School of Aerospace Medicine, Fourth Military Medical University, Xi'an, 710032, China

Running head: CTRP9 ameliorates pulmonary hypertension

[*]These authors contributed equally to this study.

[#]Correspondence to:

X. Sun, M.D., Ph. D.
Department of Pediatrics, Xijing Hospital

Fourth Military Medical University

127 Changlexi Road

Xi'an, 710032, China

E-mail: sunxin6@fmmu.edu.cn

Tel: 0086-29-84775395

Fax: 0086-29-84775395

OR

W. Xing, M.D., Ph. D.
School of Aerospace medicine
Fourth Military Medical University
169 Changlexi Road
Xi'an, 710032, China
E-mail: xwjfmmu@126.com
Tel: 0086-29-84776423
Fax: 0086-29-84776423

Figure1

A：(the blots cropped from different gels)

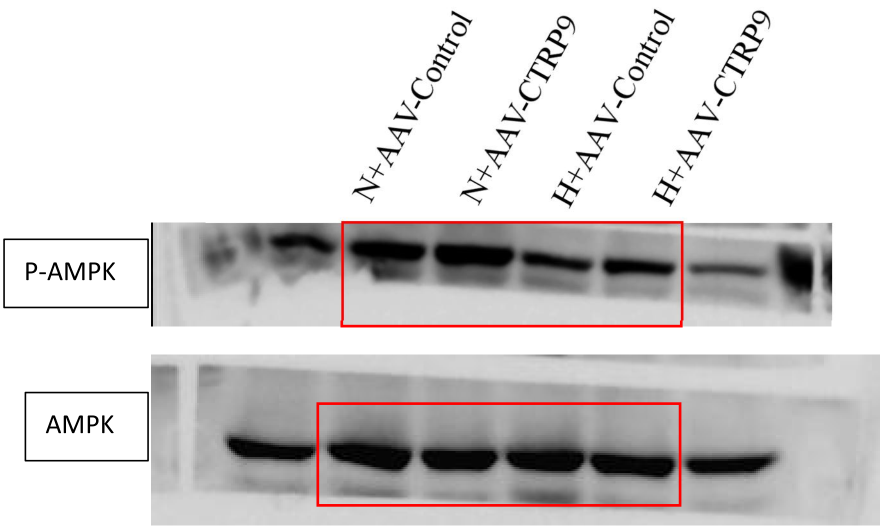

B：(the blots cropped from different gels)

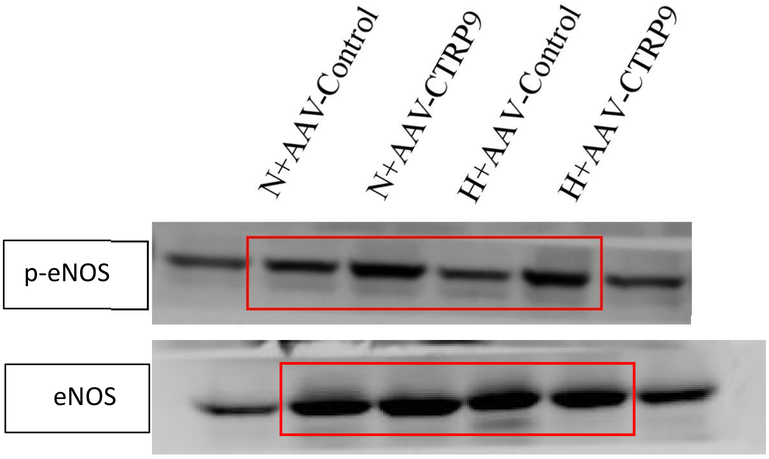

C：(the blots cropped from different gels)

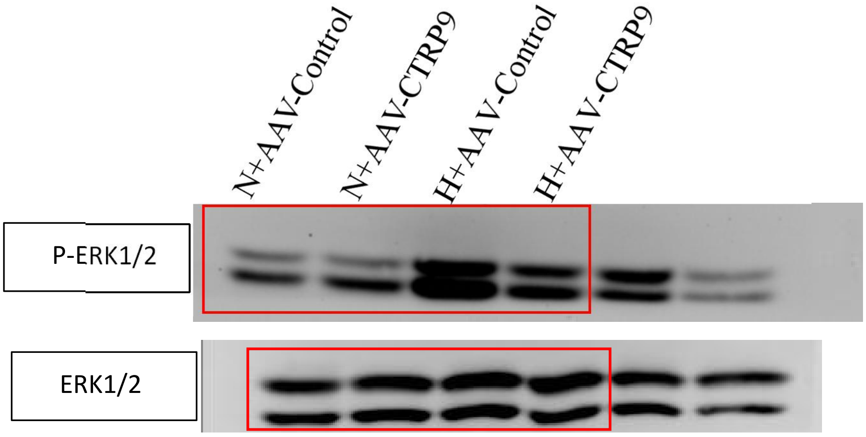
N+AAV-Control
N+AAV-CTRP9
H+AAV-Control
H+AAV-CTRP9
P-ERK1/2
ERK1/2

Figure 2

A：(the blots cropped from different part of the same gel）

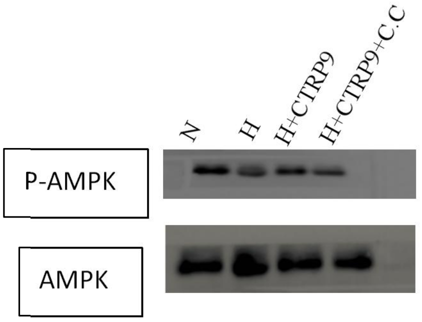

B：(the blots cropped from different gels)

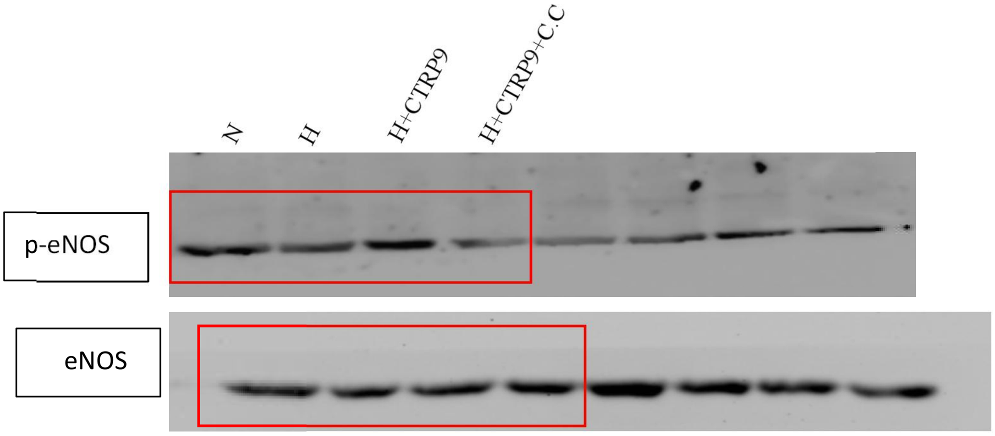

C：(the blots cropped from different gels)

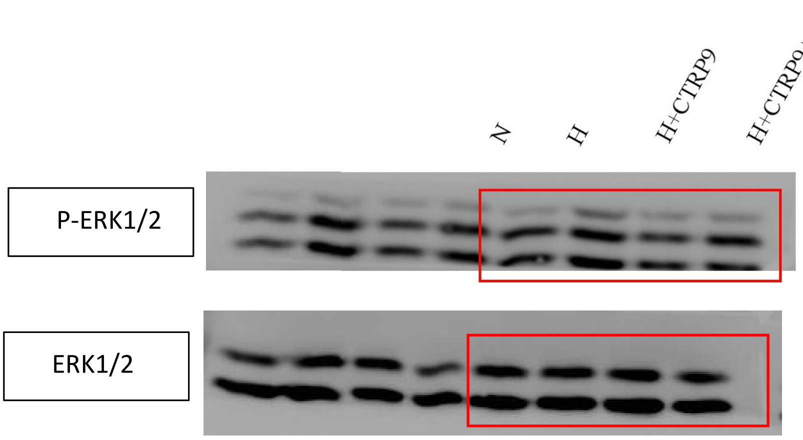

Figure 3

A：(the blots cropped from different part of the same gel）

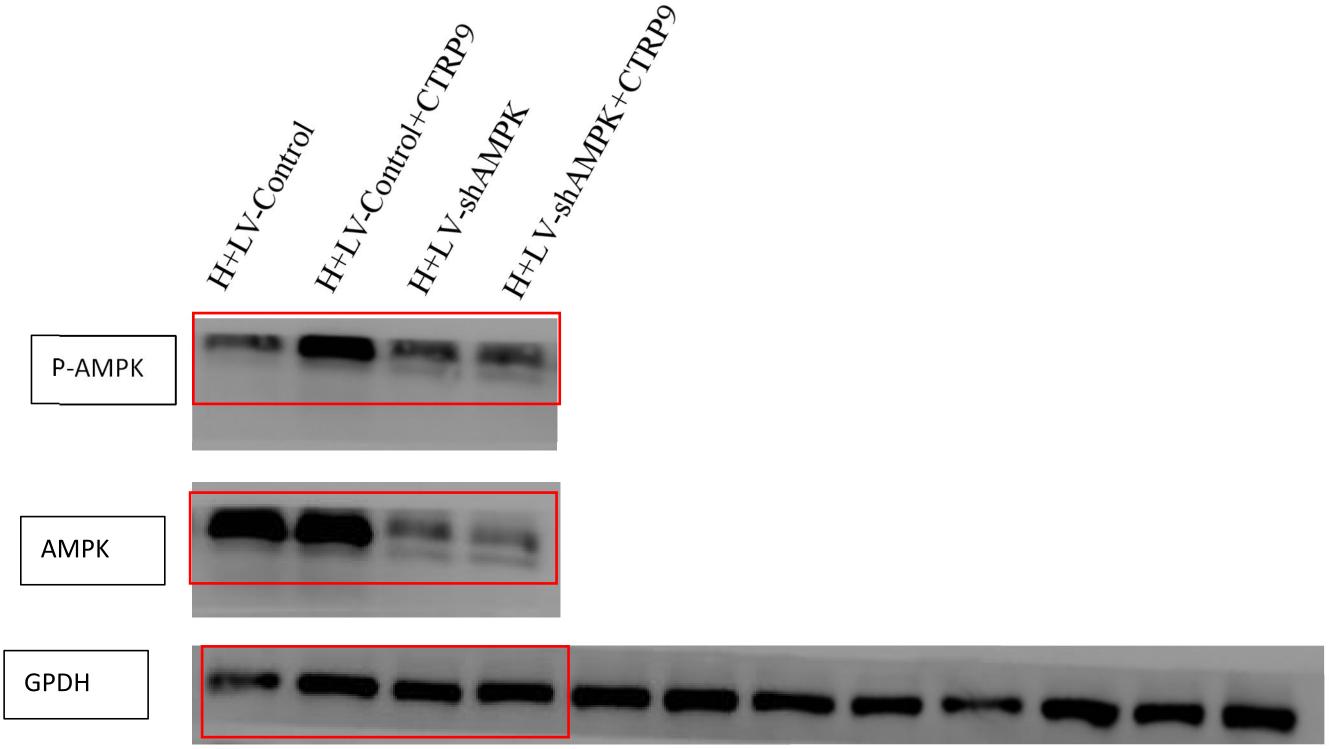

B：(the blots cropped from different gels)

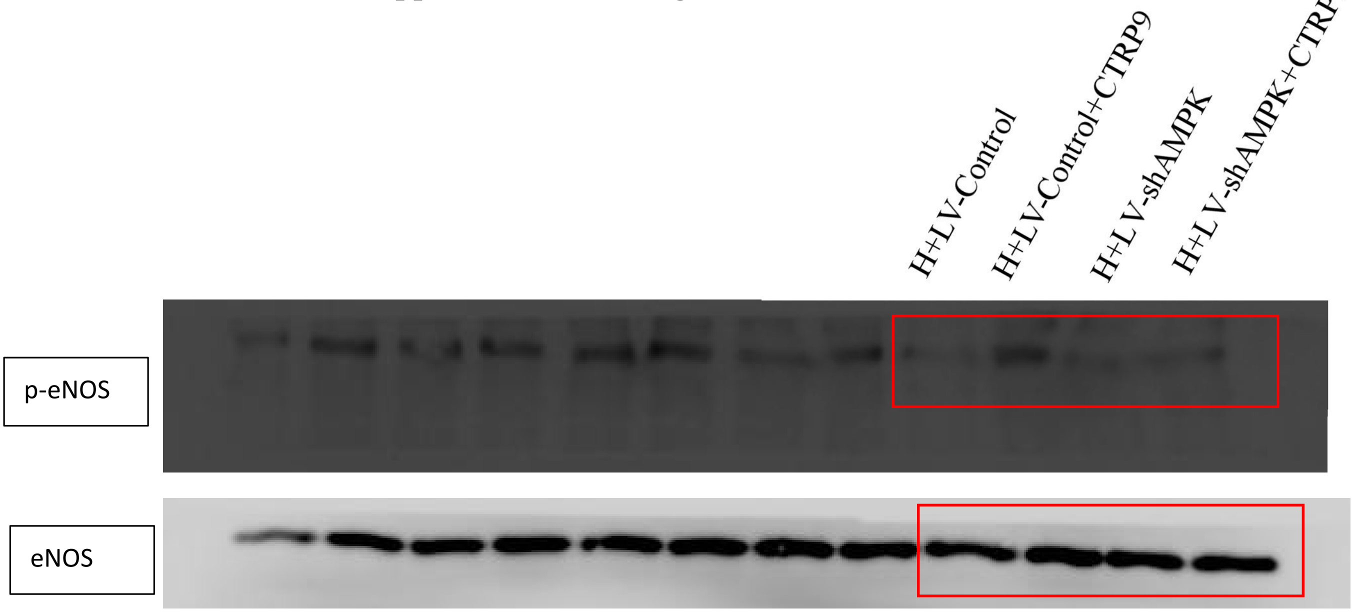

C：(the blots cropped from different gels)

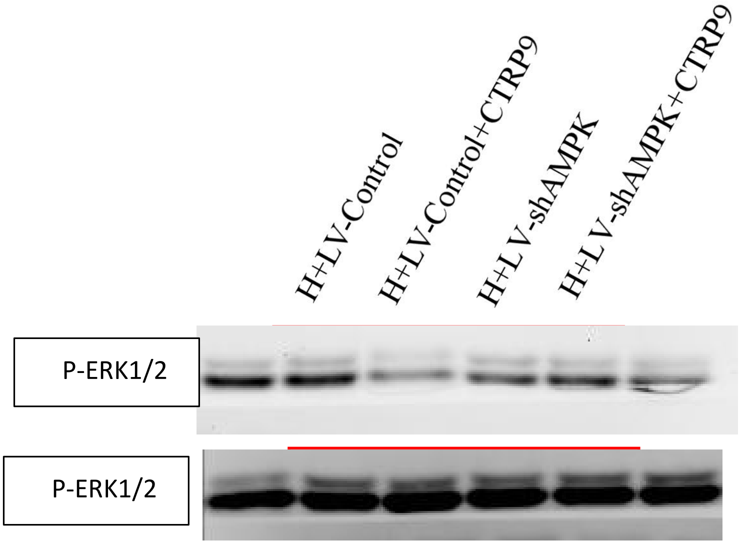

Figure 4

A：The expression of GFP in lung tissues from rat with AAV-6 intra-tracheal instillation（100×）

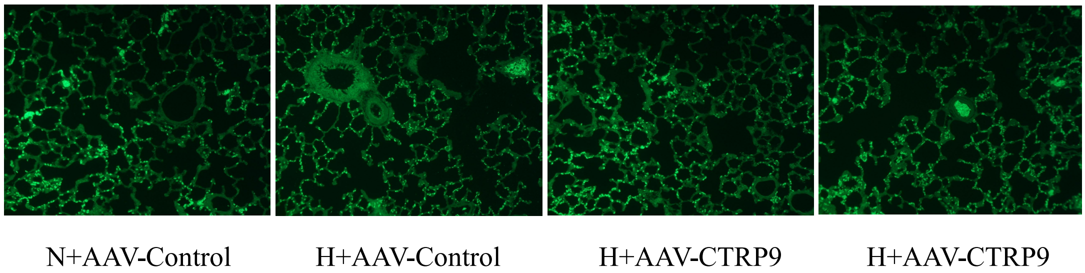

Figure 5

A: The efficacy of sh-AMPK lentivirus on AMPK expression in PMVECs

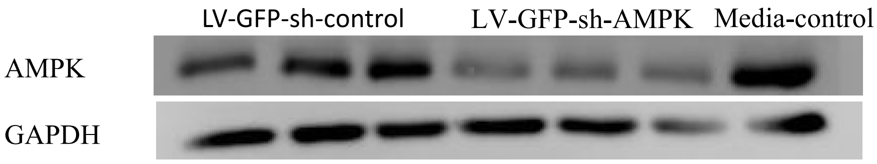

Supplement: Supplementary file 2 — Supplementary Figures. [file 41598_2021_90779_MOESM2_ESM.pdf]
